# Genome Analysis of Multi- and Extensively-Drug-Resistant Tuberculosis from KwaZulu-Natal, South Africa

**DOI:** 10.1371/journal.pone.0007778

**Published:** 2009-11-05

**Authors:** Thomas R. Ioerger, Sunwoo Koo, Eun-Gyu No, Xiaohua Chen, Michelle H. Larsen, William R. Jacobs, Manormoney Pillay, A. Willem Sturm, James C. Sacchettini

**Affiliations:** 1 Department of Computer Science and Engineering, Texas A&M University, College Station, Texas, United States of America; 2 Department of Biochemistry and Biophysics, Texas A&M University, College Station, Texas, United States of America; 3 Norman E. Bourlag Center, Texas A&M University, College Station, Texas, United States of America; 4 Howard Hughes Medical Institute, Department of Microbiology and Immunology, Albert Einstein College of Medicine, Bronx, New York, United States of America; 5 KwaZulu-Natal Research Institute for Tuberculosis and HIV, Nelson R. Mandela School of Medicine, University of KwaZulu-Natal, Durban, South Africa; University of Stellenbosch, South Africa

## Abstract

The KZN strain family of *Mycobacterium tuberculosis* is a highly virulent strain endemic to the KwaZulu-Natal region of South Africa, which has recently experienced an outbreak of extensively-drug resistant tuberculosis. To investigate the causes and evolution of drug-resistance, we determined the DNA sequences of several clinical isolates - one drug-susceptible, one multi-drug resistant, and nine extensively drug-resistant - using whole-genome sequencing. Analysis of polymorphisms among the strains is consistent with the drug-susceptibility profiles, in that well-known mutations are observed that are correlated with resistance to isoniazid, rifampicin, kanamycin, ofloxacin, ethambutol, and pyrazinamide. However, the mutations responsible for rifampicin resistance in *rpoB* and pyrazinamide in *pncA* are in different nucleotide positions in the multi-drug-resistant and extensively drug-resistant strains, clearly showing that they acquired these mutations independently, and that the XDR strain could not have evolved directly from the MDR strain (though it could have arisen from another similar MDR strain). Sequencing of eight additional XDR strains from other areas of KwaZulu-Natal shows that they have identical drug resistant mutations to the first one sequenced, including the same polymorphisms at sites associated with drug resistance, supporting the theory that this represents a case of clonal expansion.

## Introduction

Outbreaks of extensively drug-resistant (XDR) tuberculosis have become an increasing threat in certain regions around the world [1]. Though initially defined as resistance to isoniazid (INH), rifampicin (RIF), and 3 or more of the 6 classes of second-line drugs, the current definition of XDR-TB was changed by the WHO in 2006 to be: resistance to 1) INH, 2) RIF, 3) any of the fluoroquinolines, and 4) at least one of the second-line injectables (such as kanamycin, amikacin, or capreomycin). One of the most notable outbreaks was in the KwaZulu-Natal (KZN) region between 2005–2007 on the eastern seaboard of South Africa, where there is a high incidence (80%) of co-infection with HIV [2]. Among patients with TB that tested culture-positive in this region, 39% had MDR-TB, 6% had XDR-TB, and 44 out of 44 tested were HIV+. The outbreak of XDR in KwaZulu-Natal was initially reported in 2006. However, a retrospective analysis of *M. tuberculosis* isolates indicates that the first known cases of XDR-TB appeared in 2001, at the time a standard treatment regimen for MDR-TB was introduced which includes fluoroquinolones and kanamycin [3]. The XDR strain appears to be unique to this region, with a distinct genotype (by spoligotyping) referred to as F15/LAM4/KZN [3]. MIRU and RFLP analysis of several hundred other cases of XDR-TB found in other provinces in South Africa show a broad range of different genotypes, including variants of *M. tb* strain families Haarlem, EAI, LAM, and X [4].

The XDR-TB strain is considered to be at least as virulent as any other TB strain, with high mortality rates observed. For example, in the 2006 KwaZulu-Natal epidemic, 52 out of 53 patients with XDR-TB and HIV co-infection died, with a mean time from specimen collection to death of 16 days [2]. In a study of 174 patients a treatment clinic specializing in respiratory diseases in Denver, Colorado, the relative risk of death among patients with XDR-TB was found to be 7.9 times higher than for patients with MDR-TB, even with extensive treatment [5]. Similarly, in a study of HIV-negative patients in Italy and Germany, patients with XDR-TB were found to have a 5-fold higher risk of death and longer hospitalization/treatment times [6]. In Uzbekistan, 9 of 18 patients with XDR-TB died following treatment efforts [7]. However, interpretation of these results can be confounded by other factors such as untimely diagnosis and improper management. Quantitative differences in relative fitness of the XDR strain in the KZN region of South Africa (in spite of the burden of carrying mutations conferring drug resistance), as well as differences in transmissibility of this strain, remain unclear [8].

There are several hypotheses about the origin and nature of XDR strains. With bacterial infections other than TB, the appearance of drug-resistant strains is associated with chemotherapeutic usage patterns. This results from selection of naturally-occurring variants resistant to the drugs used. It has been suggested that occurrence and spread of XDR-TB in the KZN region could be related to the use of standard treatment regimens and the lack of susceptibility testing to determine appropriate chemotherapy regimens [3]. Since the F15/LAM4/KZN family of strains was already present throughout the province of KwaZulu-Natal in the susceptible as well as the MDR form in 1994 [3], the emergence of the XDR form of the strain can be explained in two ways. There could be clonal spread of one strain from one source throughout the province. This appears to have been the case for the outbreak of multi-drug resistant TB in New York City in the early 1990's, which was later shown to be a unique variant of the W-Beijing strain family [9]. Similarly, in the study in Uzbekistan, 13 of 18 patients with XDR-TB were infected with the same strain (based on DNA fingerprint), supporting the idea that a particular drug-resistant strain can achieve prevalence within a population through clonal expansion [7]. Alternatively, multiple mutational events could have taken place independently in different strains in the province, resulting in a pool of pre-existing mutants that are selected through chemotherapy [10]. Until recently, it has been assumed that drug-resistant mutants have lower fitness and tend not to spread because they will be out-competed by wild-type strains [11]. However, theoretical simulations show that even presumably “less-fit” infectious organisms can thrive within an isolated host population under the right circumstances, particularly when the drug-susceptible wild-type population is suppressed via a community-based drug-treatment program [12]. Importantly, while the clonal expansion hypothesis for the XDR outbreak in the KZN region of South Africa has been suggested in the literature [3], no experimental evidence has been presented to date to confirm this hypothesis.

The genomes of a wild-type KZN strain (KZN 4207), an MDR strain (KZN 1435), and an XDR strain have been partially sequenced at the Broad Institute (Cambridge, MA) and have been available for download since 2007 (www.broad.mit.edu/annotation/genome/mycobacterium_tuberculosis_spp). Partial analysis by Bishai's group has identified several known mutations in genes related to drug-resistance [8]. However, a thorough analysis of the differences among the strains is not possible because the MDR and XDR genome sequences are significantly incomplete. For example, they each contain ∼5000 ambiguous nucleotides ‘N’ (5,708 in KZN-1435 and 5,657 in KZN-605) and have numerous 1 bp insertions/deletions in multiple genes (e.g. *dnaB, secA, tuf*). Another indication of incompleteness is that the Broad Institute's sequence for the MDR strain (KZN-1435) has 150 short indels (<100 bp) and 46 large indels (>100 bp) relative to the wild-type strain, and the XDR sequence has 162 short indels and 37 large indels. The sequences of all three strains, including the wild-type, show a large-scale inversion of ∼2.5 Mb (spanning coordinates ∼1 Mb to ∼3.5 Mb, relative to the origin of replication). In order to validate the sequence of the wild-type strain and to determine the complete MDR and XDR sequences, we independently sequenced three KZN strains (one wild type, one MDR, and one XDR) using whole-genome sequencing. Although the wild-type strain was the same as previously sequenced (KZN 4207), we utilized the distantly-related F11 South African strain as a reference to avoid bias, completely independently from the Broad Institute's sequence. The two sequences are very similar, except that we do not find evidence for the 2.5 Mb inversion. However, our MDR and XDR genomes sequences are more complete, in contrast with those available from the Broad Institute, allowing a more accurate identification of polymorphisms relevant to drug resistance.

## Results

### Genome Sequences of Drug-Susceptible, MDR, and XDR Strains from KZN

The three strains from KwaZulu-Natal, designated KZN-V4207 (drug-susceptible), KZN-V2475 (MDR), and KZN-R506 (XDR), were obtained from patients diagnosed with pulmonary tuberculosis in 1994, 1995 and 2006 respectively (see Table 1). All patients lived in Durban, the largest city in the Kwa-Zulu-Natal region of South Africa. The first two patients were enrolled in the *Mycobacterium vaccae* vaccine trial [13], while the last patient is part of an on-ongoing study on hospital transmission of MTB. None of the patients was on anti-TB treatment at the time of sputum collection. Strain KZN-V2475 is phenotypically resistant to the first-line drugs isoniazid and rifampicin, as well as streptomycin, while strain KZN-R506 had been found to be resistant to isoniazid, rifampicin, streptomycin, kanamycin, and ofloxacin.

**Table 1:** Epidemiological data on strains sequenced in this study.

strain id	type	drug resistance^a^	year of collection	geographic location	clinic^b^	age	gender	HIV status	clincal outcome
V4207	DS	none	1995	n.a.	KGV	34 yr	female	positive	healthy
V2475	MDR	IR	1994	n.a.	KGV	24 yr	female	positive	died
R506	XDR	IRSOK	2006	Phoenix, DBN	KGV	34 yr	female	n.a.	n.a.
									n.a.
R257	XDR	IROK	2005	Tugela Ferry	KGV	38 yr	female	positive	n.a.
R262	XDR	IRESOK	2005	Durban	KEH	38 yr	female	positive	n.a.
R299	XDR	IRSOK	2005	Pomeroy	KGV	33 yr	female	positive	n.a.
R376	XDR	IROK	2005	Greytown	KGV	28 yr	female	positive	n.a.
R503	XDR	IRSOK	2005	Pietermarizburg	KGV	31 yr	female	positive	n.a.
TF274	XDR	IRSEOK	2005	n.a.	Mosvold	n.a.	n.a.	n.a.	n.a.
TF275	XDR	IRSEOK	2005	Tugela Ferry	COSH	n.a.	n.a.	n.a.	n.a.
TF490	XDR	IRSOK	2005	n.a./Mosvold	Mosvold	n.a.	n.a.	n.a.	n.a.

n.a. = not available, DS = drug susceptible.

a I = isoniazid, R = rifampicin, S = streptomycin, O = ofloxacin, K = kanamycin, E = ethambutol.

b KGV = King George V Hospital, Syndenham; KEH = King Edward VIII Hospital, Congella; COSH = Church of Scotland Hospital, Tugela Ferry.

The genomes of the three KZN strains were sequenced to >99% completion using the Illumina Genome Analyzer in so-called paired-end mode with greater than 134-fold depth of coverage. The 36-bp reads were assembled by mapping them onto the reference sequence for F11, a strain from the Western Cape of South Africa [14], and applying local contig-building to resolve apparent differences into SNPs (single nucleotide polymorphisms) and indels (insertions/deletions). The resulting (circular) genome of KZN-V4207 has 4,397,375 nucleotides and has an overall depth of coverage (average redundancy) of 134.3-fold, with a standard deviation of 41.4 (excluding the top 5% of most-highly covered sites as outliers due to redundancy). There are only 14,225 sites with a coverage of zero, mostly associated with GC-rich regions where sequencing was inefficient (9,267 sites with zero-coverage were in PGRS genes, though this represents less than 10% of the coding region spanned by this family). Analysis of the direct-repeats (DR) region indicates that KZN-V4207 has a spoligotype pattern that is missing spacers 21–24, 33–36, and 40. This is consistent with the F15/LAM4/KZN spoligotype reported by Pillay and Sturm [2].

A comparison of our sequence of KZN-V4207 to H37Rv shows that at least 973 isolated SNPs are observed (not counting those associated with indels), along with 71 small insertion/deletions (1–9 bp) and 71 medium-sized insertion/deletions (10–1000 bp). In addition, there were 28 large-scale polymorphisms (insertions or deletions >1000 bp). Many of these (15) were associated with transpositions of the IS6110 insertion elements (1355 bp repetitive elements with flanking direct repeats that contain a transposase [15]). One copy of IS1547 was also lost. The largest insertion is a 5 kb insertion in Rv2024c, which corresponds to a region containing a hypothetical protein and a cation efflux pump in other mycobacterial strains like H37Ra and F11 that had been lost in H37Rv. The largest deletion is a 9 kb region representing part of Rv1572c through Rv1587c (all annotated as hypothetical proteins); this region is also deleted in F11. Table S1 lists the locations and identities of the large-scale polymorphisms observed. When compared to the F11 genome, the KZN-V4207 sequence is observed to have fewer differences (530 SNPs, 35 small indels, and 22 large-scale indels; described in Supplemental Material), implying that they are more closely related.

The genome for KZN-V4207 has been independently sequenced at the Broad Institute (no data on depth of coverage was available). When our sequence is compared to the Broad's sequence, the sequences are identical in most functionally-significant regions. The differences are mostly associated with low-coverage regions or repetitive sequences. There are only 3 sites where the two sequences clearly disagree: a 1 bp insertion (933401 +t) near the end of an IS6110 transposon sequence, a 1 bp deletion (1277912 –g) in a 13E12 repeat, and a 1 bp deletion (1533528 –c) in PPE19. At all these sites, coverage by our reads was significantly higher using our corrected version of the genome sequence. The Broad Institute's KZN-V4207 sequence has a large-scale inversion of ∼2.5 Mb; however, this inversion was not observed in our sequencing data.

**Figure 1:**
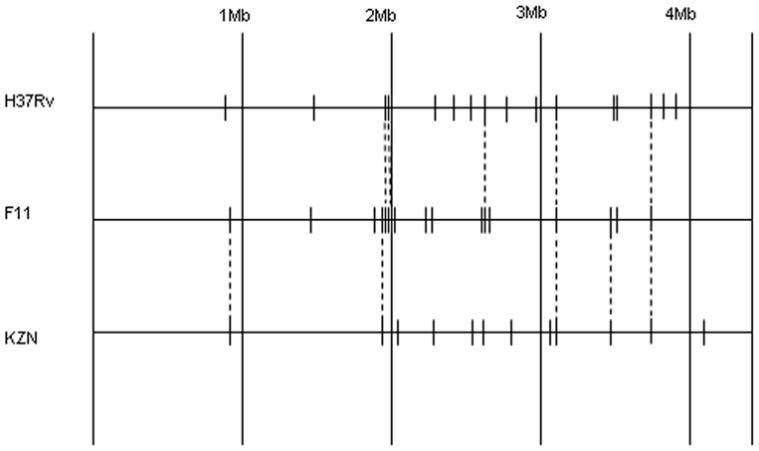
Locations of the IS-6110 insertion sequences in H37Rv, F11, and KZN-V4207. The dashed lines indicate insertion sites that have been preserved between strains. The sites are only drawn approximately to scale.

Many of the large-scale insertion/deletions among mycobacterial genomes involve IS6110 insertion sequences, which are an active family of transposons [15]. There are 16 copies of IS6110 in the H37Rv genome (see Figure 1). In F11, there are 17 copies; however many of these differ from H37Rv and represent new insertion sites. Only 5 IS6110 sites are shared in common between H37Rv and F11, 11 were excised from H37Rv, and 12 are new insertion sites in F11. By comparison, KZN-V4207 has 12 copies of IS6110. Of these 5 are the same as in F11, 6 are new insertion sites, and one (at 2.36 M) is the same as in H37Rv but is deleted in F11. Only 2 insertion sites are shared across all 3 strains (the one in the DR region, and IS6110–14 near Rv3324c/*moaC*). As previously reported, the insertion sites are accompanied by a local 3–4 bp tandem duplication that do not demonstrate any obvious sequence pattern [15]. As can be seen in Table S1, some insertions occur in non-coding regions and others occur in open-reading frames (Rv2492, Rv2775, and Rv3113 – all hypothetical genes), though none appear to occur in previously identified putative “hot-spots” [16] including the *ipl* loci [17]. There are two cases where an IS6110 sequence appears to have been deleted from one strain and re-inserted nearby in the opposite direction in KZN: one which is shared between H37Rv and KZN-V4207 (at 2.36 Mb, corresponding to IS6110-5 in H37Rv), the other being the IS6110 at 2.62 Mb that is shared with F11.

The sequences for the MDR and XDR strains were determined in a similar fashion with the Illumina GAII (in paired-end mode), except our KZN-V4207 sequence was used as the reference sequence for mapping reads. The depth of coverage was 174.7-fold and 188.3-fold, respectively. Overall, the MDR and XDR strains were found to be very similar to KZN-V4207; all three have exactly the same spoligotype. Only 67 polymorphisms were found in the MDR strain KZN-V2475, and 83 polymorphisms were localized to the XDR strain KZN-R506. Of these polymorphisms, 43 were shared between the two drug-resistant strains, relative to the drug-susceptible KZN-V4207, while 24 are unique to KZN-V2475 (MDR) and 40 are unique to KZN-R506 (XDR) (see Table 2). For cases where the MDR and XDR strains share a different relative to the wild-type, comparison of the two nucleotides to reference sequences for genomes of drug-susceptible strains (H37Rv, CDC1551, *M. tb* haarlem, and *M. bovis*) was used to determine which was the putative ancestral (non-mutated) form. This analysis shows that 29 of the 43 shared differences are unique to the two drug-resistant KZN strains, and 14 differences are unique to the KZN wild-type (where the nucleotide in the MDR and XDR strains matches the one observed in drug-susceptible strains) (see Table 2). The two drug-resistant strains also shared a 130 bp deletion in *gidB* (glucose-inhibited division protein B), relative to KZN-V4207. Tables 3, 4, 5, 6 show a selected list of mutations observed (the complete list of polymorphisms is presented in Table S2 in the Supplemental Material). The KZN-R506 (XDR) strain also has a cluster of 8 SNPs within a span of 33 base positions in *pks12*, a 4151-amino acid polyketide synthase which is involved in mycoketide biosynthesis [18]. Separately, the KZN-V2475 (MDR) strain has a cluster of 6 SNPs within 9 base positions in PPE40. The significance of these two clusters is unclear.

**Table 2:** Summary of polymorphisms observed in KZN strains.

polymorphism type	H37Rv	KZN-V4207	KZN-V2475	KZN-R506	# of sites
unique to wild-type	A	B	A	A	14
unique to MDR	A	A	B	A	24
unique to XDR	A	A	A	B	40
shared by MDR and XDR	A	A	B	B	29

‘A’ and ‘B’ indicate nucleotide identity patterns. ‘A’ represents the nucleotide in the H37Rv reference genome. An ‘A’ in the column of a KZN strain means that it matches the nucleotides at the corresponding sites in H37Rv. If a KZN strain has a ‘B’, this means it has a different nucleotide than H37Rv at those positions.

**Table 3:** Polymorphisms in the MDR strain, KZN-V2475, relative to the drug-susceptible wild-type, KZN-4207.

*gene*	*mutation*	*function*	*known resistance effect*
Rv0667 (rpoB)	D435Y	RNA-polymerase beta-subunit	rifampicin
Rv0678	M139I	transcriptional regulator	
Rv0897c	T271M	possible oxidoreductase	
Rv1940 (ribA1)	G251S	GTP cyclohydrolase (riboflavin biosynthesis)	
Rv2043c (pncA)	G132A	pyrazinamidase (nicotinamide biosynthesis)	pyrazinamide
Rv2545	P19L	hypothetical	
non-coding region	t2969973c	−150 bp upstream from Rv2645	
Rv3921c	I256V	putative membrane translocase yidC	

This table excludes synonymous mutations in coding regions, and mutations in PPE/PGRS genes or repetitive sequences. Mutations in coding regions are numbered as amino acids substitution (upper case). Numbering of base substitutions (lower case) in non-coding regions is given relative to the H37Rv genome.

**Table 4:** Polymorphisms in the XDR strain, KZN-R506, relative to the drug-susceptible wild-type, KZN-4207.

*gene*	*mutation*	*function*	*known resistance effect*
Rv0006 (gyrA)	A90V	DNA gyrase	fluoroquinolones
Rv0119 (fadD7)	I200V	fatty-acid CoA synthetase	
non-coding region	c664929a	−111 bp upstream of Rv0571c	
Rv0667 (rpoB)	D435G	RNA-polymerase beta-subunit	rifampicin
Rv0667 (rpoB)	L452P	RNA-polymerase beta-subunit	
Rv0667 (rpoB)	I1106T	RNA-polymerase beta-subunit	
Rv0849	T403I	MFS drug transporter	
non-coding region	c1272321a	−102 bp upstream of Rv1145(mmpL13a)	
Rvnr01 (rrs)	a1400g	16S ribosomal RNA	kanamycin
Rv2000	L275P	hypothetical protein	
Rv2043c (pncA)	+g in A152	pyrazinamidase (nicotinamide biosynthesis)	pyrazinamide
Rv2692 (ceoC)	S82R	TRK potassium transporter	
Rv3471c	D64E	hypothetical protein	
non-coding region	t4056430c	−55 bp upstream of Rv3616c	
Rv3806c	V188A	PRPP:decaprenyl-phosphate phosphoribosyltransferase	

This table excludes synonymous mutations in coding regions, and mutations in PPE/PGRS genes or repetitive sequences. Mutations in coding regions are numbered as amino acids substitution (upper case). Numbering of base substitutions (lower case) in non-coding regions is given relative to the H37Rv genome.

**Table 5:** Polymorphisms unique to the drug-susceptible wild-type, KZN-4207.

*gene*	*mutation*	*function*	*known resistance effect*
Rv0057	L28M	hypothetical protein	
Rv0232	+a 1 bp ins. in S127	transcription factor	
non-coding region	c713955t	−247 bp upstream of Rv0621	
Rv0691c	P61S	transcriptional regulator	
Rv1459c	P318S	membrane protein	

This table excludes synonymous mutations in coding regions, and mutations in PPE/PGRS genes or repetitive sequences. Mutations in coding regions are numbered as amino acids substitution (upper case). Numbering of base substitutions (lower case) in non-coding regions is given relative to the H37Rv genome.

**Table 6:** Polymorphisms shared between the drug-resistant strains KZN-V2475 and KZN-R506, relative to KZN-V4207, and not found in drug-susceptible strains.

*gene*	*mutation*	*function*	*known resistance effect*
Rv0103c (ctpB)	G23S	cation-transporter ATPase	
non-coding region	t1096633g	−183 bp upstream of Rv0981(mprA)	
non-coding region	t1673432a	−8 bp upstream of operon containing inhA	isoniazid
Rv1908c (katG)	S315T	catalase/peroxidase	isoniazid
Rv3926 (drrA)	R262G	membrane transporter	
Rv3795 (embB)	M306V	membrane protein, arabinosyltransferase	ethambutol
non-coding region	t4327484c	−11 upstream of ethA	ethionamide
Rv3919c (gidB)	−130 bp del, (L50–P93)	16S rRNA methyltransferase	streptomycin

This table excludes synonymous mutations in coding regions, and mutations in PPE/PGRS genes or repetitive sequences. Mutations in coding regions are numbered as amino acids substitution (upper case). Numbering of base substitutions (lower case) in non-coding regions is given relative to the H37Rv genome.

### Analysis of Mutations Related to Drug Resistance

Despite the fact that the two drug-resistant strains are clearly distinct (with 24 polymorphisms unique to the MDR strain and 40 unique to the XDR strain, as discussed above), the pattern of mutations in each is consistent with the clinical drug-susceptibility profiles of these strains. Recall that, while both strains KZN-V2475 and KZN-R506 are resistant to INH, RIF, and STR, KZN-R506 alone is also resistant to kanamycin and ofloxacin. Consistent with this, only the XDR strain KZN-R506 shows a mutation in *rrs*, the 16S rRNA, at position 1400, which explains the kanamycin resistance [19], and only the XDR strain has the A90V mutation in *gyrA* responsible for resistance to fluoroquinolones [20]. The mutation at 1400 in *rrs* is the most commonly observed mutation associated with kanamycin resistance, found in 60% of RIF-resistant clinical isolates [19]. The A90V in *gyrA* is the second-most frequently observed mutation conferring FQ resistance, found in 24% of FQ-resistant clinical isolates [21].

With respect to isoniazid (INH) resistance, both strains have the mutation of S315T in *katG*, the catalase/peroxidase that activates the pro-drug isoniazid [22]. This is the most frequently observed mutation associated with isoniazid resistance [23]. It confers high-level resistance at >5 µg/ml, compared to an MIC of 0.05 µg/ml for wild-type H37Rv [24]. Unexpectedly, both strains also have a mutation at -8 upstream of *mabA*, at the start of the operon containing the enyol-acyl-ACP reductase *inhA* (the target of the INH-NADH adduct). While mutations in this region have been reported to cause INH-resistance [23], presumably by up-regulation of expression, it is unclear why both drug-resistant strains would have this mutation, given that it is redundant with the mutation in *katG*. One possibility is that a mutation that causes a lower-level of resistance was acquired first, followed by the acquisition of a subsequent mutation that afforded a greater degree of resistance (e.g. higher MIC), enhancing fitness/survival as a isoniazid became more widely deployed in a region. Mutations at the nearby -15 position upstream of the *inhA* operon only increase the MIC several fold. However, simultaneous mutations in *katG* and the promoter region of *inhA* have previously been observed. For example, in a study of 41 clinical isolates with ETH/INH co-resistance [25], 26 were found to have the c-15t *inhA* promoter mutation, and of these, 6 also had mutations in *katG*.

Resistance to rifampicin (RIF) can be explained by mutations in *rpoB* (beta-subunit of RNA polymerase). The mutation of Asp 435 in *rpoB*, corresponding to Asp 516 in the *E. coli* numbering, has been previously observed to confer RIF resistance [26]. This is in the core 507–533 region, in which numerous mutations have been observed to cause resistance to RIF, although mutations at other sites in this region are more frequent (mutations specifically at Asp 435 constitute only 9% of all RIF-R cases). However, it is interesting to note that the two KZN strains have different mutations within the same codon, leading to different amino acid substitutions. Strain KZN-V2475 has a G->T substitution in frame 1, producing D435Y, and KZN-R506 has an A->G substitution in frame 2, producing D435G. This suggests that the two strains acquired rifampicin resistance independently. It is also notable that the XDR strain, KZN-R506, contains two additional mutations in *rpoB*, L452P and I1106T; the former (corresponding to Leu 533 in *E. coli*) is also thought to cause RIF-resistance, while the latter is not.

Streptomycin (STR) resistance is most likely due to a 130 bp deletion in *gidB* found in both drug-resistant strains (MDR and XDR), but not the wild-type. The classic STR-R mutations that have been correlated with streptomycin-resistance in the 530-loop or 915-region of *rrs*, the 16S ribosomal RNA, or in *rpsL*, the ribosomal protein S12, were not observed in either strain. However, mutations in these two genes explain only about 70% cases of STR resistance in clinical isolates [27], implying that there must be other loci that can be responsible. Recently, it was discovered by genetic mapping and comparative sequencing that mutations in *gidB* can cause low-level resistance to streptomycin in *S. coelicolor* [28]. This gene had originally been annotated as a glucose-inhibited division protein, but was later shown to be a SAM-dependent methyltransferase that methylates G527 in the 16S ribosomal RNA [28], providing an additional hydrophobic contact point in the binding site for streptomycin. A *gidB* deletion mutant increased the MIC for streptomycin from 2 µg/ml to 15 µg/ml in *E. coli*, and could be complemented by the wild-type gene [28]. Although mutations in *gidB* have previously been observed in clinical isolates of *M. tuberculosis* [28]–[30], this 130 bp deletion is distinct from every other *gidB* mutation previously reported. The 130 bp *gidB* deletion observed in the KZN MDR and XDR strains spans amino acids 50–93, which encompasses the SAM-binding site (residues 67–77) [31], and causes a frame shift for C-terminal remainder, which presumably abbrogates function completely.

Both strains also show classic mutations in *embB*, *pncA*, and the promoter region of *ethA*, which are associated with resistance to ethambutol (EMB), pyrazinamide (PZA), and ethionamide (ETH), though susceptibility to these drugs was not tested. The M306V mutation in the transmembrane protein *embB* is one of the most frequently observed mutations in EMB-resistant strains [32], putatively preventing ethambutol from interfering with biosynthesis of the arabinogalactan layer in the cell wall. In the case of *pncA*, the two drug-resistant KZN strains showed different mutations in *pncA* (a pyrazinamidase, which is thought to be involved in nicotinamide biosynthesis). The MDR strain KZN-V2475 has a G132A mutation, and mutations of this residue have previously been reported to cause resistance to PZA [33]. Strain KZN-R506 has a frame-shift mutation in amino acid 152 caused by an insertion of 1 bp, and missense mutations that cause resistance have been observed downstream of this site (so the C-terminus of the 186-residue gene product must be important). In addition, the two drug-resistant strains also share a mutation at position −8 upstream of the translational start site of *ethA*, which is a monooxygenase that activates thioamides such as ethionamide, isoxyl, and thioacetazone as pro-drugs [34]. A mutation in the upstream region could potentially confer resistance by increasing expression. However, susceptibility of the KZN strains to these drugs was not determined.

### Whole-Genome Sequencing of Additional XDR Strains

To determine whether this pattern of drug-resistance mutations was common to other XDR strains from KZN, we sequenced eight additional strains fitting the XDR profile, including clinical isolates obtained from other sites in the KZN region: TF274, R257, R503, R262, R299, TF275, R376, and TF490 (the three ‘TF’ strains were obtained from Tugela Ferry, approximately 60 miles north of Durban). All strains tested resistant to isoniazid (I), rifampicin (R), streptomycin (S), ofloxacin (O), and kanamycin (K) with the exception of strain R257, which is streptomycin-susceptible but resistant to the other four drugs. The sequencing on the Illumina GAII was performed only in single-ended mode for these strains. Reads of length 18 bp were collected for the first 5 strains, and reads of length 36 bp were collected for the last 3 strains. Despite this, we were able to get a mean depth of coverage of 24.0–41.4 over 99.8% of the genome. The KZN-V4207 wild-type sequence was used as a reference strain during sequence determination (alignment and contig-building) to reduce the possibility of bias.

When the 8 strains are put in a multiple alignment and examined for sites where there are confident differences (where two or more strains have different nucleotides, coverage >10, and homogeneity>90%), the strains appear to have nearly identical sequences. Only 5 sites (excluding those in PGRS genes and repetitive elements) met these criteria (in *nrp, fadD7, rpsK*, and two non-coding regions, sites where the base call in one or two strains differs from the others).

The bases in these 8 strains were specifically examined at 44 positions where polymorphisms were observed among the wild-type, MDR, and XDR strains (KZN-V4207, KZN-V2475, and KZN-R506, respectively) (see Table S3). The 8 strains match the base in the XDR strain 41 out of 44 positions (excepting sites in strains where there is near-zero coverage (<4) or significant heterogeneity (>30%)), regardless of whether these sites harbored MDR-specific mutations, XDR-specific mutations, or common differences in MDR and XDR relative to wild-type. One exception is the *fadD7* site mentioned above; a second polymorphic site is in *mraW* (an S-adenosyl methyl transferase), where R299 and TF275 share a ‘g’ with the XDR KZN-R506 (allowing up to 20% heterogeneity), whereas the other 6 strains share a ‘c’ with the MDR KZN-V2475 strain; a third site with a similar pattern is in Rv0849, an MFS membrane transport protein. Importantly, all 8 strains agree with the base in the XDR strain at polymorphic sites associated with drug resistance. In particular, they all show the same rifampicin-resistance mutation (D435G) in *rpoB* that distinguishes the XDR sequence from the MDR sequence (D435Y). They also share the same mutations as in both the MDR and XDR strains for ofloxacin resistance (A90V in *gyrA*), ethambutol resistance (M306V in *embB*), isoniazid resistance (S315T in *katG* and the *inhA* promoter mutation at t-8a upstream of *mabA*) and kanamycin resistance (a1400g in *rrs*). The R257 strain appears to contain the 130 bp deletion in *gidB* like the other strains, despite the fact that it was reported to be streptomycin-susceptible. It is possible that the drug susceptibility was mis-characterized, due to the low-level of resistance that *gidB* mutations have been observed to confer [28]. The strain was unavailable for re-testing at lower concentrations.

## Discussion

Based on the sequences of these KZN strains, it appears that multi-drug resistance results from a combination of previously-observed mutations, each responsible for resistance to a different drug through classic mechanisms/targets. This suggests that there is no common underlying cause of drug-resistance to multiple drugs, such as broad-specificity drug efflux pump getting over-expressed, or a mutation in a gene that changes the permeability of the mycobacterial cell wall [26]. The MDR and XDR strains contain typical mutations in *gyrA, rpoB, rrs, katG*, and the promoter of *inhA* that explain resistance to fluroquinalones, rifampicin, kanamycin, and isoniazid. Although susceptibilities to ethambutol and pyrazinamide were not determined clinically, mutations in *embB* and *pncA* were observed as well. The fact that the MDR and XDR strains have different mutations in *rpoB* and *pncA* suggests that they arose separately, and that these mutations were acquired independently after divergence. This observation argues against the hypothesis that the XDR strain might have evolved directly from the MDR strain (though it could have arisen from another similar MDR strain). While resistance to streptomycin is usually associated with mutations in *rpsL* or *rrs*, the KZN MDR and XDR strains showed a rare 130 bp deletion in *gidB*. Although recent studies have begun to show that mutations can cause low-level resistance to streptomycin, through abbrogation of ribosomal methylation, this mutation is unique and has never been reported before.

The observation that the eight additional extensively drug-resistant strains isolated from the KwaZulu-Natal region in South Africa all appear to be nearly identical to KZN-R506 strongly suggests that the epidemic of drug-resistant tuberculosis in KwaZulu-Natal represents a clonal expansion of the same strain. That these drug-resistant strains can thrive and attain high-frequency in the population despite the heavy burden of carrying so many drug-resistance mutations could be potentially due to 1) lower fitness cost than typically assumed, 2) offset of fitness cost by increased virulence due to some other as yet unidentified mutation(s), or 3) side-effects of drug treatment practices in the region that leads to the suppression of the wild-type drug-susceptible strain locally and selection for the drug-resistant strain(s). Another possibility is that spread of these strains could be associated with diminished immunity in the host, since most of the patients from which samples were obtained were HIV-positive. Further analysis and comparison of the genome sequences we have reported could lead to a better understanding of the nature of the virulence of this strain.

## Methods

Approval by an Institutional Review Board was not required for this study, as no patient-specific data was reported. Nor was patient consent required to publish genome sequences of bacterial samples.

### Drug Susceptibility Testing

The susceptibility of the clinical isolates was tested on solid media using standard protocols [3] for drugs at the following concentrations: isoniazid, 1 mg/L; rifampicin, 1 mg/L; ethambutol, 7.5 mg/L; streptomycin, 2 mg/L; ofloxacin, 2 mg/L; kanamycin, 5 mg/L.

### DNA Preparation and Sequencing Reaction

Sequencing of the genomes of three KZN strains, KZN-V4207, KNZ-V2475, and KZN-R506, was carried out on the Illumina Genome Analyzer II (Illumina). In this study, Illumina Paired-End sequencing method (PE) was used. The cetyltrimethylammonium bromide (CTAB)-lysozyme method was used for extraction and purification of genomic DNA [35]. DNA samples were prepared for the GAII as described on the sample preparation protocol (Illumina). 2–3 µg of genomic DNA was initially used for sample preparation. Genomic DNA was sheared by a nebulizer to generate DNA fragments for the Illumina Paired-End Sequencing method. The specific oligonucleotides (Illumina adapters) designed for PE sequencing were ligated to both ends of DNA fragments with the TA cloning method. Adapter-ligated DNA fragments of length 350–400 bp were isolated from a 2% agarose gel (Certified low-range Ultra Agarose, BIO-RAD) by using QIAquick Gel Extraction Kit (QIAGEN). Then the fragments were amplified by PCR reaction to generate the DNA library (15–30 ng/µl). The median size of the library was estimated by examining the 2% agarose gel image. The molarity of the DNA library was estimated as described on the sample preparation protocol (Illumina): MW of the library  =  S (Median size of the library) x 650. The DNA libraries (5 pM) including φX control (bacteriophage DNA) were loaded on the flow cell for the cluster generation and sequencing. 72 cycles of images were collected, representing pairs of 36-bp reads. The images were analyzed using version 0.3 of the GAPipeline software supplied by Illumina, producing files with ∼30 million of pairs of 36 bp reads for each genome (28.0 M reads for KZN-V4207, 27.1 M for KZN-V2475 and 31.2 M for KZN-R506).

### Sequence Determination and Bioinformatics

The reads were analyzed by comparative genome assembly to determine the complete sequence of each genome using custom software developed in our lab. The reads were first mapped against the reference sequence for the genome of the F11 South African strain. The mapping was accomplished by identifying the position(s) in the genome that each fragment (including its reverse complements) matches with no gaps and at most 2 mismatches. Initially the reads in each pair were treated as independent; subsequently, mapped locations of reads for which the paired-end did not match within 300 bp were discarded. The mapped reads were used to assemble a list of the nucleotides observed at each position within the reference genome contributed by all the reads that overlapped it. Base calls were made by a maximum likelihood calculation, computed as the product of the probabilities for each base at each position using uncertainties estimated during image analysis. Sites where apparent differences were observed were subjected to local contig-building (described in more detail in Supplemental Methods in Supporting Online Material S1), in order to determine whether the difference was due to a nucleotide substitution or a small insertion or deletion. Larger-scale deletions were identified by analyzing paired-end data for reads whose paired-end maps an unusually long distance away (i.e. >300 bp, compared to average read length of ∼200). Large-scale insertions were determined by identifying reads not mapping into the F11 genome that significantly cover regions in other mycobacterial genomes.

For each genome, a list of contig-verified differences is prepared and used to modify the reference genome to produce an intermediate (‘edited’) genome. Then the process is repeated by re-mapping the reads against the edited genome, and re-calling bases at each position. For any sites that still had 0 coverage, the sequence from the reference strain was used. This included most of the PGRS genes, which have exceptionally-high GC-content (80–90%). In addition, any putative SNPs in low-coverage sites where the majority base differed from the reference genome but the majority consisted of only 1 or 2 bases were rejected due to lack of sufficient data, and replaced with the base in the reference genome. Information on each individual site is available as a supplement to the genome sequence itself (using an expanded sequence file format that indicates the called nucleotide, coverage, quality, and other information for each position).

Virtual spoligotyping was performed by aligning (without gaps) all the reads obtained for each KZN strain against each of the 43 spacer sequences (26-bp oligos) from the direct repeats (DR) regions [36]. The number of matching reads for each spacer was counted, considering both forward and reverse-complement sequences, and accepting up to 1 nucleotide mismatch. Spacers with 0 matches were interpreted as missing.

Whole-genome alignments of the KZN sequences to other mycobacterial strains were generated using MUMMER version 3.20 [37].

This Whole Genome Shotgun project has been deposited at DDBJ/EMBL/GenBank under the project accession numbers ACVS00000000 (KZN 4207), ACVT00000000 (KZN V2475), and ACVU00000000 (KZN R506). The full-length genome sequences in FASTA format are provided in the Supplementary Material, along with the inferred annotation (table of protein coding regions) based on F11 (Table S4, S5, S6, S7, S8, S9).

## Supporting Information

Table S1 Large-scale polymorphisms between KZN-V4207 and H37Rv. Sequence positions are given relative to H37Rv.
(0.03 MB DOC)

Table S2 A complete list of the SNPs found among KZN-V4207 (wt), KZN-V2475 (MDR), and KZN-R506 (XDR). Also shown are bases at the corresponding sites for H37Rv and F11. These positions were selected as those sites at which either the MDR or XDR strain differed from the wild-type. The depth of coverage and purity (percentage of bases corresponding to the majority) are also shown. Information on the amino acid mutation and known relationships to drug resistance are shown. The mutations are grouped into those that are MDR-specific, XDR-specific, or found in the wild-type strain only. NCR = non-coding region.
(0.52 MB DOC)

Table S3 A list of nucleotides found in XDR strains TF274, R257, R503, R262, R299, TF275, R376, and TF490 at sites of polymorphism among KZN-V4207 (drug susceptible), KZN-V2475 (MDR) and KZN-R506 (XDR), excluding those in PPE and PGRS genes, and repetitive regions. Sites with low coverage (<4) or heterogeneity (>30%) are marked with a ‘?’. Sites where there are differences among the 8 XDR strains are marked with a ‘*’.
(0.03 MB DOC)

Table S4 Full-length genome sequence of KZN 4207 in FASTA format. Nucleotides from the reference genome F11 were used to fill in regions with zero coverage.
(4.46 MB TXT)

Table S5 Inferred annotation of KZN 4207 protein coding regions based on alignment to F11, in tab-separated ASCII format.
(0.38 MB TXT)

Table S6 Full-length genome sequence of KZN V2475 in FASTA format. Nucleotides from the reference genome F11 were used to fill in regions with zero coverage.
(4.46 MB TXT)

Table S7 Inferred annotation of KZN V2475 protein coding regions based on alignment to F11, in tab-separated ASCII format.
(0.39 MB TXT)

Table S8 Full-length genome sequence of KZN R506 in FASTA format. Nucleotides from the reference genome F11 were used to fill in regions with zero coverage.
(4.46 MB TXT)

Table S9 Inferred annotation of KZN R506 protein coding regions based on alignment to F11, in tab-separated ASCII format.
(0.38 MB TXT)

Supporting Online Material S1 (0.05 MB DOC)
