# Supplementary material for: Genome Analysis of Multi- and Extensively-Drug-Resistant Tuberculosis from KwaZulu-Natal, South Africa
Source: PLoS One. 2009 Nov 5;4(11):e7778. doi: 10.1371/journal.pone.0007778 (PMC2767505; doi:10.1371/journal.pone.0007778)
Supplement: Table S1 — Large-scale polymorphisms between KZN-V4207 and H37Rv. Sequence positions are given relative to H37Rv. (0.03 MB DOC) [file pone.0007778.s001.doc]

Insertions:

locus position length disrupts identity

L91 334653 2842 PGRS3 PGRS region in F11 but lost from H37Rv

L238 932204 1358 non-coding IS6110

L484 2047372 1358 non-coding IS6110

L542 2268725 5000 Rv2042c contains hypothetical protein and cation efflux pump from H37Ra

L573 2366898 1045 non-coding IS6110

L614 2555931 1358 non-coding IS6110

L626 2634119 3442 non-coding IS6110 + esat-6 + PPE from H37Ra

L656 2807873 1358 Rv2492 IS6110

L721 3083207 1359 Rv2775 IS6110

L798 3480373 1358 Rv3113 IS6110

L896 3934699 2532 PGRS54 PGRS region in F11 but lost from H37Rv

L937 4077861 1358 non-coding IS6110

Deletions:

locus position length identity

L120 453367 2606 Rv0376c-Rv0378

L223 889021 1359 IS6110

L372 1541952 1358 IS6110

L425 1779243 9247 Rv1572c-Rv1585c; hypoth. proteins; also deleted in F11

L470 1986626 1077 Rv1754c-Rv1755c/plcD and part of Rv1758/cut1

L572 2365415 1358 IS6110

L589 2430117 1358 IS6110

L613 2550014 1358 IS6110

L651 2784613 1358 IS6110

L698 2972109 1358 IS6110

L809 3551230 1358 IS6110

L810 3552713 1358 IS6110

L835 3710382 2149 IS6110+IS1547

L860 3795058 1358 IS6110

L871 3842289 4926 Rv3425/PPE57-Rv3429/PPE59, includes IS1532

L883 3890779 1358 IS6110
