# Supplementary material for: Genome Analysis of Multi- and Extensively-Drug-Resistant Tuberculosis from KwaZulu-Natal, South Africa
Source: PLoS One. 2009 Nov 5;4(11):e7778. doi: 10.1371/journal.pone.0007778 (PMC2767505; doi:10.1371/journal.pone.0007778)
Supplement: Table S2 — A complete list of the SNPs found among KZN-V4207 (wt), KZN-V2475 (MDR), and KZN-R506 (XDR). Also shown are bases at the corresponding sites for H37Rv and F11. These positions were selected as those sites at which either the MDR or XDR strain differed from the wild-type. The depth of coverage and purity (percentage of bases corresponding to the majority) are also shown. Information on the amino acid mutation and known relationships to drug resistance are shown. The mutations are grouped into those that are MDR-specific, XDR-specific, or found in the wild-type strain only. NCR = non-coding region. (0.52 MB DOC) [file pone.0007778.s002.doc]

|  |  | H37Rv | H37Rv | F11 | KZN-4207 | coverage | purity | V2475 | coverage | purity | R506 | coverage | purity | mutation | synon | drug resistance | function |
| --- | --- | --- | --- | --- | --- | --- | --- | --- | --- | --- | --- | --- | --- | --- | --- | --- | --- |
| **MDR-only** | |  |  |  |  |  |  |  |  |  |  |  |  |  |  |  |  |
| Rv0278c | PGRS3 | 333683 | A | A | A | 5 | 0.60 | **C** | 4 | 1.00 | A | 10 | 0.80 | G876G | synon |  |  |
| Rv0667 | rpoB | 761109 | G | G | G | 116 | 0.98 | **T** | 176 | 1.00 | G | 147 | 0.99 | D435Y |  | RIF | RNA polymerase beta |
| Rv0678 | - | 779406 | G | G | G | 138 | 0.99 | **A** | 206 | 0.96 | G | 195 | 0.99 | M139I |  |  | transcription factor |
| Rv0897c | - | 1001604 | G | G | G | 140 | 0.98 | **A** | 239 | 0.99 | G | 217 | 1.00 | T271M |  |  | oxidoreductase |
| Rv0980c | PGRS18 | 1095661 | G | A | A | 0 | 0.00 | **G** | 3 | 1.00 | A | 0 | 0.00 | A264A | synon |  |  |
| Rv0980c | PGRS18 | 1095859 | G | G | G | 5 | 0.80 | **C** | 3 | 1.00 | G | 7 | 0.71 | A198G |  |  |  |
| NCR | Rep034 | 1276938 | G | G | G | 126 | 1.00 | **C** | 171 | 0.93 | G | 188 | 1.00 | A215P |  |  | repetitive sequence |
| Rv1450c | PGRS27 | 1633077 | A | A | A | 10 | 0.80 | **C** | 16 | 0.62 | A | 13 | 0.46 | G517G | synon |  |  |
| Rv1468c | PGRS29 | 1656150 | G | G | G | 3 | 0.67 | **C** | 3 | 1.00 | G | 1 | 1.00 | A191G |  |  |  |
| Rv1940 | ribA1 | 2193248 | G | G | G | 91 | 1.00 | **A** | 157 | 0.99 | G | 149 | 0.99 | G215S |  |  | riboflavin biosynthesis |
| Rv2043c | pncA | 2288847 | C | C | C | 170 | 0.99 | **G** | 230 | 0.99 | C | 217 | 1.00 | G132A |  | PZA | pyrazinamidase |
| Rv2356c | PPE40 | 2638432 | T | T | T | 151 | 1.00 | **G** | 180 | 1.00 | T | 139 | 0.99 | G368G | synon |  |  |
| Rv2356c | PPE40 | 2638433 | C | C | C | 153 | 1.00 | **T** | 180 | 1.00 | C | 141 | 0.99 | G368E |  |  |  |
| Rv2356c | PPE40 | 2638434 | C | C | C | 153 | 0.99 | **T** | 185 | 0.99 | C | 138 | 1.00 | G368R |  |  |  |
| Rv2356c | PPE40 | 2638435 | A | A | A | 153 | 0.99 | **G** | 189 | 0.96 | A | 141 | 0.99 | T367T | synon |  |  |
| Rv2356c | PPE40 | 2638436 | G | G | G | 154 | 0.99 | **T** | 191 | 1.00 | G | 147 | 0.99 | T367N |  |  |  |
| Rv2356c | PPE40 | 2638441 | T | T | T | 163 | 0.99 | **G** | 185 | 1.00 | T | 153 | 0.99 | S365S |  |  |  |
| Rv2383c | mbtB | 2674578 | G | G | G | 109 | 0.99 | **C** | 160 | 1.00 | G | 149 | 1.00 | P420P | synon |  | mycobactin biosynthesis |
| Rv2545 | - | 2867838 | C | C | C | 209 | 1.00 | **T** | 190 | 1.00 | C | 173 | 1.00 | P19L |  |  | hypothetical protein |
| Rv2591 | PGRS44 | 2922944 | A | A | A | 5 | 0.60 | **G** | 12 | 0.50 | A | 7 | 0.71 | D465G |  |  |  |
| NCR |  | 2969973 | T | T | T | 128 | 1.00 | **C** | 214 | 1.00 | T | 210 | 1.00 |  |  |  | -150bp upstream from Rv2645 |
| Rv3259 | - | 3639574 | G | G | G | 135 | 0.99 | **A** | 173 | 0.99 | G | 133 | 1.00 | P50P | synon |  |  |
| Rv3345c | PGRS50 | 3739067 | A | A | A | 10 | 1.00 | **C** | 6 | 0.50 | A | 11 | 0.91 | G1236G | synon |  |  |
| Rv3921c | yidC | 4409304 | T | T | T | 130 | 0.98 | **C** | 118 | 0.97 | T | 104 | 1.00 | I256V |  |  | translocase |
|  |  |  |  |  |  |  |  |  |  |  |  |  |  |  |  |  |  |
| **XDR-only** |  |  |  |  |  |  |  |  |  |  |  |  |  |  |  |  |  |
| Rv0006 | gyrA | 7570 | C | C | C | 240 | 0.99 | C | 236 | 1.00 | **T** | 222 | 1.00 | A90V |  | OFL | DNA gyrase |
| Rv0119 | fadD7 | 144646 | A | A | A | 139 | 0.98 | A | 171 | 1.00 | **G** | 143 | 0.83 | I200V |  |  | acyl-CoA synthetase |
| NCR |  | 664929 | C | C | C | 190 | 0.99 | C | 212 | 0.99 | **A** | 232 | 1.00 |  |  |  | -111bp upstream of Rv0571c |
| Rv0663 | atsD | 756757 | C | C | C | 123 | 0.98 | C | 214 | 0.99 | **T** | 148 | 0.99 | Y207Y | synon |  | aryl sulfatase |
| Rv0667 | rpoB | 761110 | A | A | A | 116 | 0.98 | A | 180 | 0.99 | **G** | 139 | 1.00 | D435G |  | RIF | RNA polymerase beta |
| Rv0667 | rpoB | 761161 | T | T | T | 80 | 0.97 | T | 103 | 0.97 | **C** | 107 | 0.99 | L452P |  |  | RNA polymerase beta |
| Rv0667 | rpoB | 763123 | T | T | T | 189 | 0.99 | T | 228 | 0.98 | **C** | 186 | 0.99 | I1106T |  |  | RNA polymerase beta |
| Rv0849 | - | 947263 | C | C | C | 122 | 0.98 | C | 133 | 0.98 | **T** | 125 | 0.85 | T403I |  |  |  |
| Rv0980c | PGRS18 | 1095855 | A | A | C | 3 | 1.00 | C | 1 | 1.00 | **A** | 6 | 0.83 | G199G | synon |  |  |
| Rv1087 | PGRS21 | 1212153 | T | T | T | 0 | 0.00 | T | 0 | 0.00 | **C** | 3 | 1.00 | G198G | synon |  |  |
| Rv1091 | PGRS22 | 1217053 | C | C | C | 5 | 1.00 | C | 1 | 1.00 | **G** | 3 | 1.00 | G195G | synon |  |  |
| NCR |  | 1272321 | C | C | C | 147 | 0.99 | C | 181 | 0.99 | **A** | 179 | 1.00 |  |  |  | -102bp upstream of Rv1145(mmpL13a) |
| Rv1196 | PPE18 | 1339432 | G | C | C | 87 | 0.85 | C | 46 | 0.67 | **G** | 181 | 0.99 | A28A | synon |  |  |
| Rv1196 | PPE18 | 1339435 | T | G | G | 92 | 0.92 | G | 45 | 0.73 | **T** | 197 | 0.98 | A29A | synon |  |  |
| Rv1196 | PPE18 | 1339436 | C | A | A | 92 | 0.97 | A | 48 | 0.71 | **C** | 194 | 0.98 | Q30Q | synon |  |  |
| Rv1197 | esxK | 1340830 | G | G | G | 140 | 0.94 | G | 207 | 0.50 | **A** | 291 | 1.00 | A58T |  |  | repetitive region, esat-6 family |
| Rvnr01 | rrs | 1473246 | A | A | A | 230 | 1.00 | A | 276 | 1.00 | **G** | 199 | 1.00 | S467S |  | KAN |  |
| NCR | Rep044 | 1633662 | G | G | G | 4 | 1.00 | G | 1 | 1.00 | **C** | 3 | 1.00 | H377D |  |  | repetitive sequence |
| NCR | Rep045 | 1637408 | G | G | G | 1 | 1.00 | G | 0 | 0.00 | **A** | 3 | 1.00 | R329W |  |  | repetitive sequence |
| NCR | Rep058 | 2196715 | G | C | C | 128 | 0.95 | C | 231 | 0.94 | **G** | 176 | 1.00 | V243V | synon |  | repetitive sequence |
| Rv2000 | - | 2246032 | T | T | T | 209 | 1.00 | T | 245 | 0.98 | **C** | 222 | 1.00 | L275P |  |  | hypothetical protein |
| Rv2043c | pncA | 2288788 | - | - | - | 0 | 0.00 | - | 0 | 0.00 | **G** | 224 | 1.00 |  |  | PZA | +1bp ins, frame shift |
| Rv2048c | pks12 | 2295692 | C | C | C | 92 | 1.00 | C | 221 | 0.58 | **T** | 202 | 1.00 | V3765V | synon |  |  |
| Rv2048c | pks12 | 2300546 | A | T | T | 82 | 1.00 | T | 146 | 0.99 | **A** | 133 | 0.99 | H2147H | synon |  |  |
| Rv2048c | pks12 | 2300666 | G | G | G | 110 | 1.00 | G | 150 | 0.97 | **A** | 132 | 0.98 | D2107D | synon |  |  |
| Rv2048c | pks12 | 2300669 | G | G | G | 105 | 1.00 | G | 144 | 0.99 | **C** | 132 | 1.00 | P2106P | synon |  |  |
| Rv2048c | pks12 | 2300674 | T | T | T | 99 | 1.00 | T | 140 | 1.00 | **C** | 131 | 1.00 | N2105D |  |  |  |
| Rv2048c | pks12 | 2300676 | T | T | T | 99 | 1.00 | T | 138 | 1.00 | **A** | 133 | 0.99 | Y2104F |  |  |  |
| Rv2048c | pks12 | 2300678 | T | T | T | 101 | 0.98 | T | 141 | 0.99 | **C** | 132 | 0.97 | L2103L | synon |  |  |
| Rv2048c | pks12 | 2300680 | G | G | G | 97 | 0.98 | G | 147 | 0.96 | **A** | 129 | 1.00 | L2103L | synon |  |  |
| Rv2048c | pks12 | 2300690 | G | G | G | 83 | 0.98 | G | 144 | 0.99 | **A** | 133 | 1.00 | D2099D | synon |  |  |
| Rv2048c | pks12 | 2300699 | G | G | G | 79 | 0.97 | G | 144 | 0.99 | **A** | 123 | 1.00 | R2096R | synon |  |  |
| Rv2165c | mraW | 2428457 | C | C | C | 143 | 1.00 | C | 232 | 1.00 | **G** | 209 | 0.81 | R271R | synon |  |  |
| Rv2692 | ceoC | 3010269 | C | C | C | 148 | 0.99 | C | 247 | 0.99 | **G** | 56 | 0.55 | S82R |  |  | TRK potassium transporter |
| Rv3343c | PPE54 | 3732517 | A | A | A | 180 | 1.00 | A | 150 | 0.62 | **G** | 153 | 1.00 | I1473I | synon |  |  |
| Rv3343c | PPE54 | 3732525 | A | A | A | 170 | 1.00 | A | 133 | 0.58 | **T** | 156 | 1.00 | F1471I |  |  |  |
| Rv3343c | PPE54 | 3732553 | A | A | A | 167 | 0.94 | A | 143 | 0.52 | **G** | 171 | 0.99 | I1461I | synon |  |  |
| Rv3471c | - | 3889150 | G | G | G | 160 | 0.99 | G | 186 | 0.99 | **T** | 181 | 0.99 | D64E |  |  | mannose-6-phosphate isomerase |
| NCR |  | 4056430 | T | T | T | 239 | 1.00 | T | 259 | 1.00 | **C** | 258 | 1.00 |  |  |  | -55bp upstream of Rv3616c |
| Rv3806c | - | 4269271 | A | A | A | 146 | 0.99 | A | 221 | 0.99 | **G** | 185 | 0.99 | V188A |  |  | phosphoribosyltransferase |
|  |  |  |  |  |  |  |  |  |  |  |  |  |  |  |  |  |  |
| **shared by DR** | |  |  |  |  |  |  |  |  |  |  |  |  |  |  |  |  |
| Rv0020c | TB39.8 | 24125 | G | G | G | 126 | 0.99 | **A** | 189 | 1.00 | **A** | 169 | 1.00 | L440L | synon |  |  |
| Rv0103c | ctpB | 122107 | C | C | C | 148 | 0.99 | **T** | 226 | 1.00 | **T** | 196 | 0.98 | G23S |  |  | cation transporter |
| Rv0746 | PGRS9 | 836897 | T | T | T | 5 | 0.80 | **G** | 5 | 0.80 | **G** | 5 | 0.80 | G399G | synon |  |  |
| NCR |  | 1096633 | T | G | T | 252 | 1.00 | **G** | 229 | 0.70 | **G** | 207 | 1.00 |  |  |  | -183bp upstream of Rv0981(mprA) |
| Rv1067c | PGRS19 | 1189921 | C | C | C | 21 | 0.71 | **T** | 46 | 1.00 | **T** | 40 | 1.00 | G168G | synon |  |  |
| Rep034 | REP-4 | 1276882 | C | C | C | 129 | 1.00 | **G** | 226 | 1.00 | **G** | 191 | 1.00 | A196G |  |  |  |
| Rep034 | REP-4 | 1276885 | C | C | C | 129 | 0.99 | **T** | 221 | 1.00 | **T** | 205 | 1.00 | P197L |  |  |  |
| Rv1197 | esxK | 1340916 | G | G | G | 74 | 0.99 | **A** | 295 | 1.00 | **A** | 273 | 1.00 | E86E | synon |  |  |
| Rv1361c | PPE19 | 1532777 | T | T | T | 132 | 1.00 | **C** | 143 | 0.99 | **C** | 156 | 0.99 | Q286R |  |  |  |
| NCR |  | 1673432 | T | T | T | 100 | 1.00 | **A** | 142 | 0.98 | **A** | 138 | 0.99 |  |  | INH | inhA promoter: T-8A |
| NCR | Rep048 | 1789516 | A | G | A | 114 | 0.96 | **G** | 149 | 0.99 | **G** | 156 | 1.00 |  |  |  | repetitive sequence |
| Rv1908c | katG | 2155168 | C | G | C | 117 | 0.97 | **G** | 178 | 1.00 | **G** | 198 | 0.99 | S315T |  | INH | katG: S315T |
| Rv2141c | - | 2401402 | G | G | G | 137 | 0.98 | **T** | 223 | 0.99 | **T** | 213 | 1.00 | G107G | synon |  |  |
| Rv2936 | drrA | 3272997 | A | A | A | 224 | 1.00 | **G** | 230 | 0.99 | **G** | 188 | 0.99 | R262G |  |  | drug efflux pump |
| Rv3343c | PPE54 | 3730466 | A | A | A | 196 | 0.64 | **G** | 177 | 0.58 | **G** | 161 | 0.99 | I2157T |  |  |  |
| Rv3345c | PGRS50 | 3741286 | - | C | C | 2 | 1.00 | **T** | 3 | 1.00 | **T** | 7 | 1.00 |  |  |  |  |
| Rv3345c | PGRS50 | 3741587 | A | A | A | 4 | 0.75 | **C** | 6 | 0.83 | **C** | 8 | 0.62 | G396G | synon |  |  |
| NCR | Rep173 | 3883683 | G | G | G | 95 | 0.99 | **C** | 115 | 0.93 | **C** | 118 | 0.90 | W45S |  |  | repetitive sequence |
| NCR | Rep173 | 3883686 | T | T | T | 103 | 0.99 | **G** | 118 | 0.98 | **G** | 120 | 1.00 | V46G |  |  |  |
| NCR | Rep173 | 3883690 | G | G | G | 104 | 1.00 | **A** | 135 | 0.99 | **A** | 134 | 1.00 | T47T | synon |  |  |
| NCR | Rep173 | 3883707 | T | T | T | 135 | 1.00 | **C** | 211 | 0.98 | **C** | 180 | 0.99 | L53S |  |  |  |
| NCR | Rep173 | 3883711 | G | G | G | 145 | 1.00 | **A** | 220 | 1.00 | **A** | 203 | 0.99 | T54T | synon |  |  |
| NCR | Rep173 | 3883845 | T | T | T | 115 | 1.00 | **C** | 169 | 1.00 | **C** | 148 | 1.00 | V99A |  |  |  |
| NCR | Rep173 | 3883915 | G | G | G | 119 | 1.00 | **A** | 206 | 1.00 | **A** | 203 | 1.00 | T122T | synon |  |  |
| NCR |  | 4120983 | A | A | A | 87 | 0.62 | **G** | 171 | 0.50 | **G** | 171 | 0.54 |  |  |  | repetitive region, 28bp downstream of Rv3680 |
| Rv3795 | embB | 4247429 | A | A | A | 111 | 0.99 | **G** | 159 | 1.00 | **G** | 122 | 0.98 | M306V |  | EMB | arabinosyl transferase |
| NCR |  | 4327484 | T | T | T | 133 | 1.00 | **C** | 219 | 0.95 | **C** | 197 | 0.96 |  |  | ETH | ethA promoter: T-11C |
| Rv3919c | gidB | 4407922 | A | A | A | 136 | 1.00 | **-** | - | - | **-** | - | - |  |  | STR | -130bp deletion, RNA methyltransferase |
| Rv3921c | yidC | 4409995 | G | G | G | 170 | 0.99 | **A** | 195 | 1.00 | **A** | 161 | 1.00 | Y25Y | synon |  | translocase |
|  |  |  |  |  |  |  |  |  |  |  |  |  |  |  |  |  |  |
| **unique to wt** | |  |  |  |  |  |  |  |  |  |  |  |  |  |  |  |  |
| Rv0057 | - | 59977 | C | C | **A** | 192 | 0.98 | C | 217 | 1.00 | C | 199 | 0.99 | L28M |  |  |  |
| Rv0232 | - | 278278 | - | - | **A** | 154 | 0.99 | - | 0 | 0.00 | - | 0 | 0.00 |  |  |  | +1bp frame shift in transcription factor |
| Rv0507 | mmpL2 | 598344 | G | G | **A** | 229 | 1.00 | G | 214 | 1.00 | G | 181 | 1.00 | V382V | synon |  |  |
| NCR |  | 713955 | C | C | **T** | 104 | 1.00 | C | 147 | 1.00 | C | 147 | 1.00 |  |  |  | -247 upstream of Rv0621 |
| Rv0691c | - | 791486 | G | G | **A** | 211 | 1.00 | G | 276 | 1.00 | G | 260 | 1.00 | P61S |  |  |  |
| Rv1415 | ribA2 | 1591662 | C | C | **T** | 170 | 0.99 | C | 181 | 1.00 | C | 163 | 1.00 | G422G | synon |  | riboflavin biosynthesis |
| Rv1459c | - | 1645187 | G | G | **A** | 108 | 1.00 | G | 158 | 1.00 | G | 137 | 0.99 | P318S |  |  | integral membrane protein |
| Rv2383c | mbtB | 2674095 | G | G | **A** | 60 | 1.00 | G | 66 | 1.00 | G | 77 | 1.00 | A581A | synon |  |  |
| Rep085 | IS1081'-4 | 2983095 | A | G | **G** | 262 | 0.79 | A | 168 | 0.53 | A | 175 | 0.99 |  |  |  |  |
| Rv2866 | - | 3177968 | G | G | **A** | 129 | 1.00 | G | 212 | 0.98 | G | 191 | 0.99 | L49L | synon |  |  |
| Rv3350c | PPE56 | 3762124 | A | A | **G** | 197 | 1.00 | A | 246 | 1.00 | A | 234 | 1.00 | I1660T |  |  |  |
| Rv3478 | PPE60 | 3894625 | C | T | **T** | 127 | 0.54 | C | 138 | 0.99 | C | 155 | 0.96 | A67V |  |  |  |
| Rv3703c | - | 4146850 | C | C | **T** | 96 | 0.98 | C | 132 | 1.00 | C | 127 | 1.00 | A14A | synon |  |  |
| Rv3870 | - | 4348127 | C | C | **T** | 147 | 1.00 | C | 210 | 0.99 | C | 185 | 0.98 | T549T | synon |  |  |
