# Supplementary material for: Genome Analysis of Multi- and Extensively-Drug-Resistant Tuberculosis from KwaZulu-Natal, South Africa
Source: PLoS One. 2009 Nov 5;4(11):e7778. doi: 10.1371/journal.pone.0007778 (PMC2767505; doi:10.1371/journal.pone.0007778)
Supplement: Table S3 — A list of nucleotides found in XDR strains TF274, R257, R503, R262, R299, TF275, R376, and TF490 at sites of polymorphism among KZN-V4207 (drug susceptible), KZN-V2475 (MDR) and KZN-R506 (XDR), excluding those in PPE and PGRS genes, and repetitive regions. Sites with low coverage (<4) or heterogeneity (>30%) are marked with a ‘?’. Sites where there are differences among the 8 XDR strains are marked with a ‘*’. (0.03 MB DOC) [file pone.0007778.s003.doc]

H37Rv |V4207 V2475 R506 |TF274 R257 R503 R262 R299 TF275 R376 TF490

(DS) |(DS) (MDR) (XDR) |(XDR) (XDR) (XDR) (XDR) (XDR) (XDR) (XDR) (XDR)

----- |----- ----- ----- |----- ----- ----- ----- ----- ----- ----- -----

MDR-only mutations

------------------

Rv0667 rpoB 761109 G | G T G | G G G G G G G G

Rv0678 - 779406 G | G A G | G G G G G G G G

Rv0897c - 1001604 G | G A G | G G G G G G G G

Rv1940 ribA1 2193248 G | G A G | ? G G G G G G G

Rv2043c pncA 2288847 C | C G C | C C C C C C C C

Rv2545 - 2867838 C | C T C | C C C C C C C C

non coding 2969973 T | T C T | T T T T T T T T

Rv3259 - 3639574 G | G A G | G G G G G G G G

Rv3921c - 4409304 T | T C T | T T T T T T T T

XDR-only mutations

------------------

Rv0006 gyrA 7570 C | C C T | ? T ? T T T T T

Rv0119 fadD7 144646 A | A A G | A A A A G * ? A A

non coding 664929 C | C C A | A A ? A A A A A

Rv0663 atsD 756757 C | C C T | ? T T T T T T T

Rv0667 rpoB 761110 A | A A G | ? G ? G G G G G

Rv0667 rpoB 761161 T | T T C | C C ? C C ? C ?

Rv0667 rpoB 763123 T | T T C | C C ? C C C C C

Rv0849 - 947263 C | C C T | C C C C T * T * C C

non coding 1272321 C | C C A | A A ? A A A A A

Rvnr01 rrs 1473246 A | A A G | G ? ? G G G G G

Rv2000 - 2246032 T | T T C | C C ? C C C C C

Rv2048c pks12 2300546 A | T T A | ? A ? A A ? ? ?

Rv2165c mraW 2428457 C | C C G | C C C C G * G * C C

Rv3471c - 3889150 G | G G T | T ? ? T ? T T T

non coding 4056430 T | T T C | C C ? C C C C C

Rv3806c - 4269271 A | A A G | ? G ? G ? G G G

mutations in MDR and XDR

------------------------

Rv0020c TB39.8 24125 G | G A A | A ? ? A ? A A A

Rv0057 - 59977 C | A C C | C C C C C C C C

Rv0103c ctpB 122107 C | C T T | T T ? T T T T T

Rv0507 mmpL2 598344 G | A G G | G G ? G G G G G

non coding 713955 C | T C C | C C C C C C C C

Rv0691c - 791486 G | A G G | G G G G G G G G

Rv1415 ribA2 1591662 C | T C C | C C C C C C C C

Rv1459c - 1645187 G | A G G | G G G G G G G G

non coding 1673432 T | T A A | A A A A A A A A

Rv1908c katG 2155168 C | C G G | G G ? G G G G G

Rv2141c - 2401402 G | G T T | T T ? T T T T T

Rv2866 - 3177968 G | A G G | G G G G G G G G

Rv2936 drrA 3272997 A | A G G | G G ? G G G G G

Rv3703c - 4146850 C | T C C | C C C C C C C C

Rv3795 embB 4247429 A | A G G | G G G G G G G G

non coding 4327484 T | T C C | C C ? C C C C C

Rv3870 - 4348127 C | T C C | C C C C C C C C

Rv3921c - 4409995 G | G A A | A A ? A A A A A
