# Supplementary material for: Genome Analysis of Multi- and Extensively-Drug-Resistant Tuberculosis from KwaZulu-Natal, South Africa
Source: PLoS One. 2009 Nov 5;4(11):e7778. doi: 10.1371/journal.pone.0007778 (PMC2767505; doi:10.1371/journal.pone.0007778)
Supplement: Supporting Online Material S1 — (0.05 MB DOC) [file pone.0007778.s010.doc]

Supporting Online Material

**Methods**

When sequencing in paired-end mode, 72 cycles of images were collected from the Illumina GAII Genome Analyzer. The images were analyzed using version 0.3 of the GAPipeline software supplied by Illumina, producing millions of associated pairs of 36-bp reads. The reads were analyzed by comparative genome assembly to determine the complete sequence of each genome using custom software developed in our lab. The reads were first mapped against the reference sequence for the genome of the F11 South African strain. The mapping was accomplished by identifying the position(s) in the genome that each fragment (including its reverse complements) matches with no gaps and at most 2 mismatches. Initially the reads in each pair were treated as independent; subsequently, mapped locations of reads for which the paired-end did not match within 300 bp were discarded. The mapped reads were used to assemble a list of the nucleotides observed at each position within the reference genome contributed by all the reads that overlapped it.

The base called at each position was determined by calculating the likelihood of each of the bases and choosing the base with the maximum score. The likelihood is calculated as the product of the probabilities of each base *b* at each position *i*, over all the reads *j* covering that position*,*

*p(b|i)=Π_j_* *p(b|j)*,

where *p(b|j)* is the probability of base *b* in read *j* at the position corresponding to *i* in the genome. These probabilities are extracted from the quality scores *Q* output by the image analysis software via *p=1-1/(1+10^Q/10^)*, as described in the Illumina documentation. This probabilistic procedure almost always results in calling the same base as the majority, but is more accurate by taking into account uncertainty in the image analysis.

Differences between the observed base and the expected base, along with any sites with coverage less than 20, were subjected to local contig building. In this procedure, a read perfectly matching the genome 50-100 bp upstream and another matching 50-100 bp downstream of a site are selected. Then a hash table is constructed from reads whose paired-end maps to within 500 bp of this region. The hash table indexes the reads (by prefixes of length 16), in both forward and reverse-complement directions, for fast lookup. Then a best-first search is performed to find a sequence of perfectly overlapping reads that connects from the starting read to the ending read. During the search, partial contigs are prioritized by maximum degree of overlap, to minimize the shifts between adjacent reads (typically 1-2 bp, but with a maximum cutoff of 15). If a contig can be constructed that connects to the read downstream, then the contig is aligned against the region in the reference genome using standard dynamic programming. The alignment reveals whether sites with observed differences from the reference genome are actually single-nucleotide polymorphisms (SNPs) or insertion or deletions (indels).

Larger-scale deletions were identified by analyzing reads whose paired end maps an unusually long distance away. The mean distance between the mapped locations of the ends of fragments was 196.7 with standard deviation 41.8 (for KZN-V4207). Reads whose paired-end mapped more than two standard deviations farther away than the usual fragment length (e.g. dist>300) were clustered and used to identify places where a possible deletion occurred, which was subsequently verified by contig-building (to determine the precise boundaries). An attempt was made to build contigs from all the remaining reads after removing those that could be mapped into the F11 genome, but the contigs obtained could not be interpreted (via a BLAST search). However, the non-F11 reads were also mapped against the reference sequence for the H37Rv genome to identify other large-scale insertions of mycobacterial DNA that had been deleted from the F11 genome. Two large insertions were identified this way: 1100 bp spanning Rv1334-Rv1336, and 2330 bp covering PE2, an IS6110 sequence, and PE36. In addition, the region spanning spacers 9-11 (lost from F11) was added back in based on the sequence in H37Rv, and verified by regaining high coverage through the portion of the direct repeats region.

For each genome, a list of differences verified by contig-building is prepared and used to modify the reference genome to produce an intermediate (‘edited’) genome. Then the process is repeated by re-mapping the reads against the edited genome, and re-calling bases at each position. For any sites that still had 0 coverage, the sequence from the reference strain was used. This included regions in most of the PGRS genes, which have exceptionally-high GC-content (80-90%). In addition, any putative SNPs in low-coverage sites where the majority base differed from the reference genome but the majority consisted of only 1 or 2 bases were rejected due to lack of sufficient data, and replaced with the base in the reference genome.

The three genomes were aligned to each other, to the three publicly-available KZN strains sequenced at the Broad Institute, and to other reference genomes, using MUMMER version 3.20 (Kurtz et al., 2004), to determine which SNPs and indels are shared by other drug-susceptible or drug-resistant strains. Comparison to the sequence of KZN-V4207 determined at the Broad Institute revealed 105 polymorphic loci. Of these, 20 were deemed to be mistakes in our initial sequence determination, where there were apparent SNPs or low-coverage in our sequence but where we had been unable to build contigs to correct these regions. Since mapping of our reads against the Broad’s sequence in these regions resulted in higher coverage, these regions were corrected in our final sequence for KZN-V4207 using the Broad’s sequence. However, there were 85 other loci where differences between our sequence and the Broad Institute’s sequence were attributed to low sequence coverage (our sequencing data was insufficient to confirm either sequence) or ambiguity (where our data supported both sequence alternatives with high coverage), further described in the main text. These sites remain as specified in F11 (for low-coverage sites) or as called (for ambiguous sites), and are indicated in the supplement to the sequence file.

For sequencing the 8 additional KZN XDR strains, TF274, R257, R503, R262, R299, TF275, R376, and TF490, a similar procedure as described above was followed for whole-genome sequencing on the Illumina Genome Analyzer, though in single-ended mode. However, due to equipment-malfunction during the middle of the run, only reads of 18-bp in length could be collected for the first 5 strains. As a consequence, SNPs could be observed at well-covered sites reliably, but indels could not be resolved by local contig-building. The reads were mapped against the sequence of the KZN wild-type strain KZN-V4207 (to avoid biasing toward mutations in the MDR or XDR strains), and the bases at each position were tabulated. The mean depth of coverage for the first 5 strains ranged between 23.6 (TF274) and 52.6 (R299). The base at each position was called using the maximum likelihood formula given above. Only bases at positions showing polymorphisms among the first three stains (KZN-V4207, KZN-V2475, and KZN-R506) were analyzed. The bases observed at these sites are shown in Table S3.

**Comparison Between the KZN-4207 and F11 Genomes**

When compared to the F11 genome, the sequence of the KZN-V4207 genome has 530 isolated SNPs, 35 small indels (length 1-9 bp), 18 medium-sized indels (45-472 bp), and 22 large-scale insertions and deletions (>1000 bp). KZN-V4207 has three large regions (re-)inserted which were deleted from F11 relative to H37Rv: 1100 bp spanning Rv1334-Rv1336, 2330 bp covering a non-coding region, PE2, an IS6110 sequence and part of PPE36, and 221 bp spanning spacers 9-11 in the direct repeats region. In terms of large-scale deletions, KZN-V4207 has a deletion of 322 bp in Rv0145, a deletion of 2606 bp covering Rv0376c-Rv0378, a deletion of 472 bp in a non-coding region including the stop codon of *ddl*), a deletion of 7345 bp covering Rv3424c-Rv3427, a 9kb region including *plcD* that had been spanned by two IS6110 sequences (leaving only one copy, see below), and a deletion of 2818 bp covering IS1547, Rv3327 and Rv3328c (*sigJ*). In addition, there is a loss of 74 bp in the direct repeats region corresponding to spacer 40 and an adjacent copy of a direct repeat, and an insertion of 221 bp corresponding to the gain of the region spanning spacers 9-11 (which had been lost in F11, relative to H37Rv).

**Comparison to KZN Genome Sequences Determined at the Broad Institute**

The genome for KZN-V4207 has been independently sequenced at the Broad Institute, and is available for download from www.**broad**.mit.edu/annotation/genome/mycobacterium_tuberculosis_spp/**KZN**Downloads.html. When our sequence is compared to the Broad's sequence, the sequences are identical in most functionally-significant coding regions. There were 85 loci where differences were observed, comprised of 36 SNPs (or clusters of SNPs) and 49 indels. However, almost all of these fall into two categories: 43 are in low-coverage regions (0-4x), where nucleotides from F11 had been used by default (42 out of these 43 loci were in PGRS genes), and 42 other loci involve regions where *both* sequence versions are supported by our data (both have high-coverage, >25x), due to ambiguity in highly-redundant regions (e.g. repetitive elements, as well as highly-duplicated families such as esat-6 and PPE proteins). Only two potentially legitimate differences were detected (a 1 bp deletion of a G in an 13E12 repeat-family protein, and a 1 bp deletion of a C in PPE19), where the coverage is significantly higher with these indels than without. The Broad Institute's KZN-V4207 sequence has a large-scale inversion of ~2.5M bp in the middle of the chromosome; however, evidence for this inversion was not observed in our sequencing data.

**References**

Kurtz S, Phillippy A, Delcher AL, Smoot M, Shumway M (2004). Versatile and open software for comparing large genomes. *Genome Biol*, 5(2):R12.
